# Supplementary material for: A Valence-Bond-Based Multiconfigurational Density Functional Theory: The λ-DFVB Method Revisited
Source: Molecules. 2021 Jan 20;26(3):521. doi: 10.3390/molecules26030521 (PMC7863953; doi:10.3390/molecules26030521)
Supplement: Supplementary file 1 [file molecules-26-00521-s001.pdf]

A Valence Bond-Based Multiconfigurational Density Functional Theory: The λ-DFVB Method Revisited

Peikun Zheng [1], Chenru Ji [1], Fuming Ying [1], Peifeng Su [1,*] and Wei Wu [1,*]

[1] Fujian Provincial Key Laboratory of Theoretical and Computational Chemistry, The State Key Laboratory of Physical Chemistry of Solid Surfaces, College of Chemistry and Chemical Engineering, Xiamen University, Xiamen, China

* Correspondence: supi@xmu.edu.cn; weiwu@xmu.edu.cn

SUPPLEMENTARY INFORMATION

Figure S1: Potential energy curves for H_2.

Figure S2: Potential energy curves for F_2.

Figure S3: Potential energy curves for HF.

Figure S4: Potential energy curves for N_2.

Figure S5: Potential energy curves for C_2.

Table S1: Comparison of different indexes and λ values of diatomic molecules in equilibrium distances.

Table S2: Comparison of different indexes and λ values of the molecules in the AE6 dataset.

Table S3: Comparison of different indexes and λ values of several atoms in the second row.

Table S4: Comparison of different indexes and λ values of the transition states in DBH24 dataset.

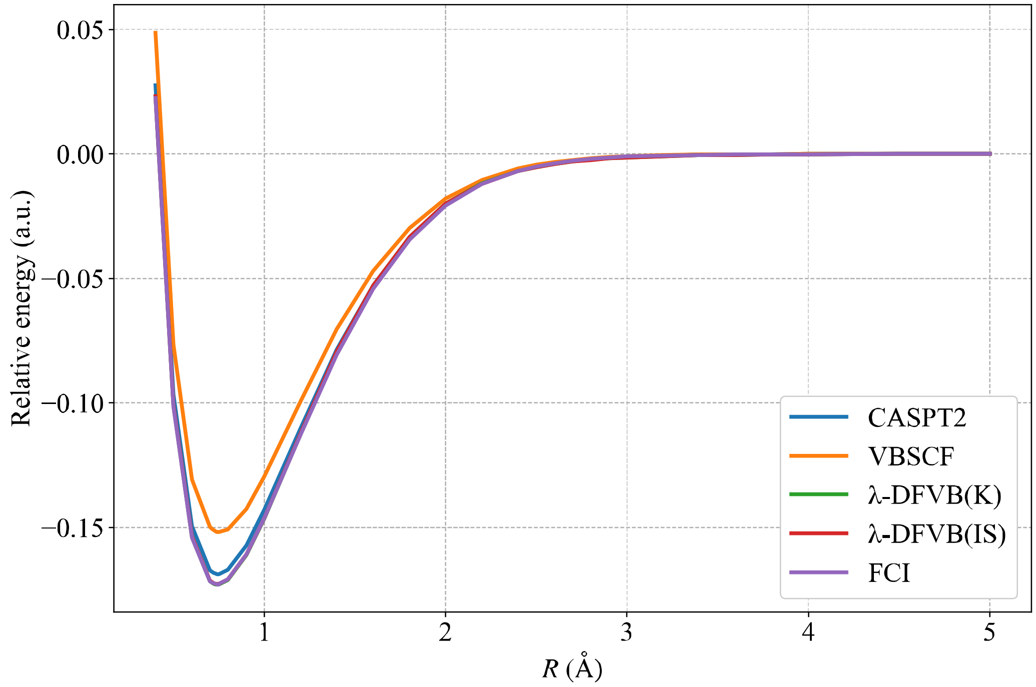

Figure S1: Potential energy curves for H_2.

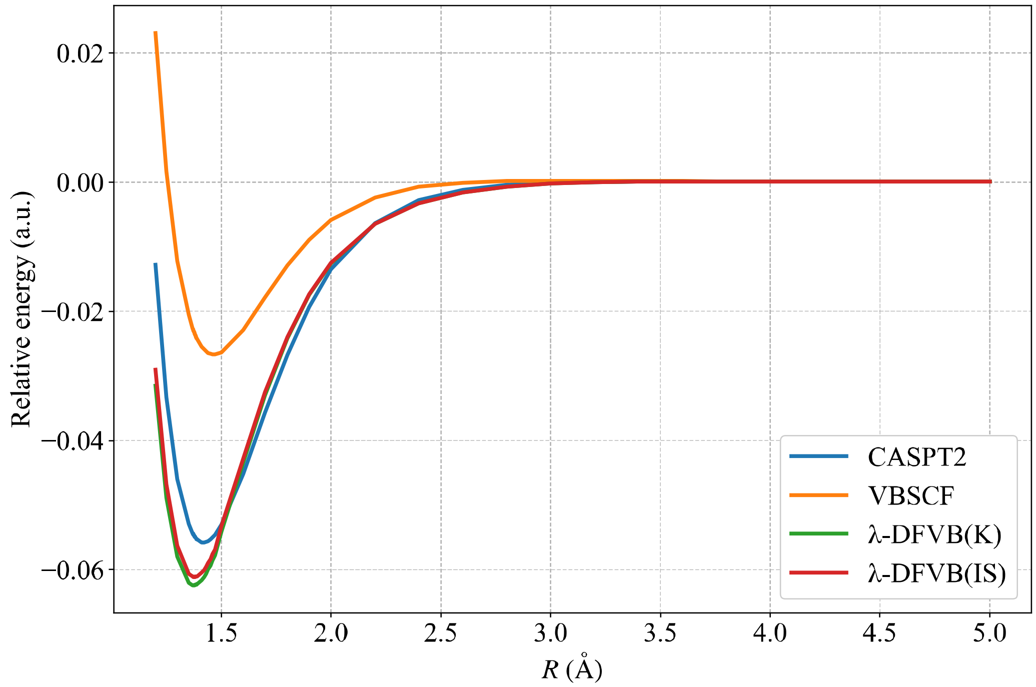

Figure S2: Potential energy curves for F_2.

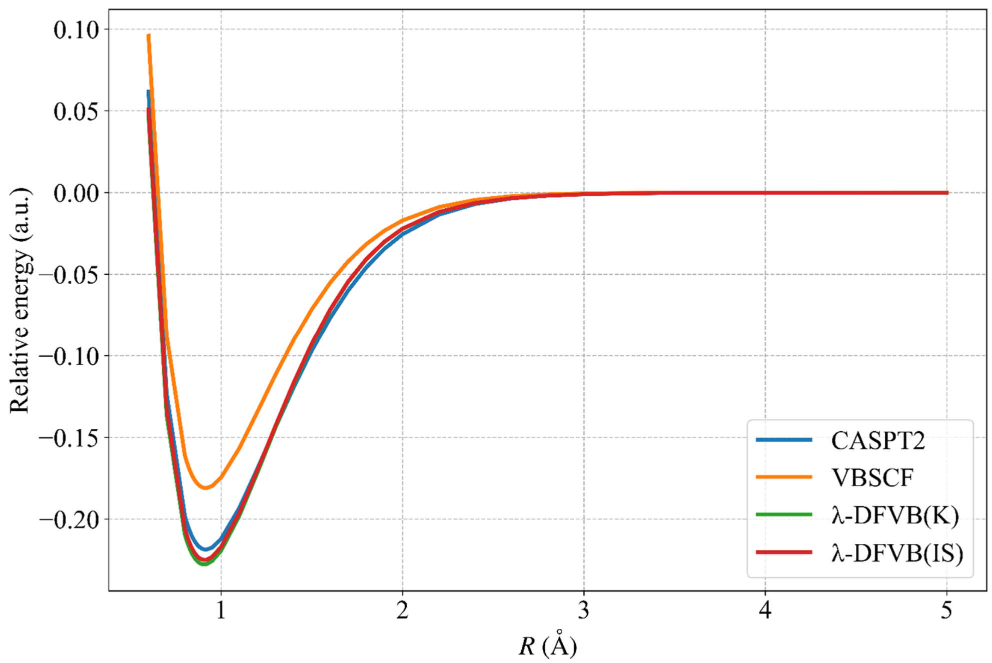

Figure S3: Potential energy curves for HF.

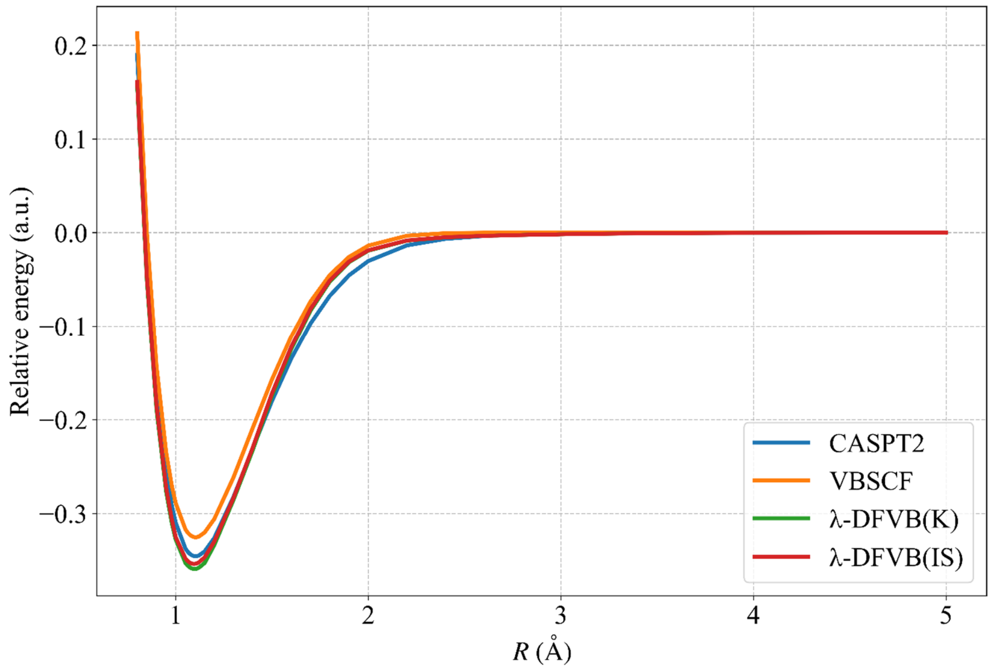

Figure S4: Potential energy curves for N_2.

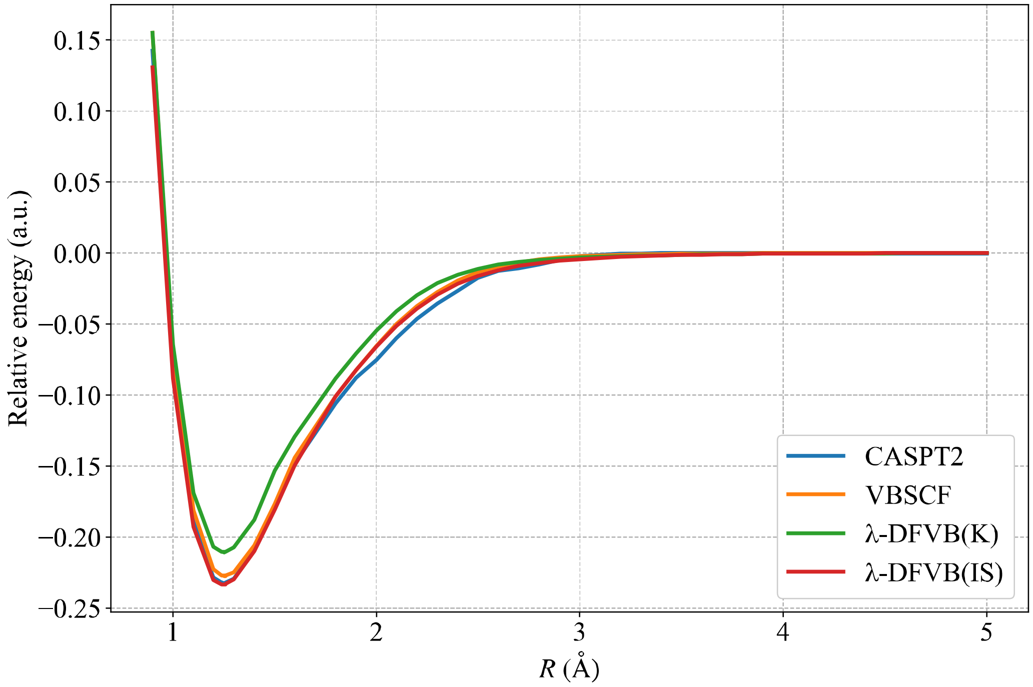

Figure S5: Potential energy curves for C_2.

Table S1. Comparison of different indexes and λ values of diatomic molecules in equilibrium distances.

	S_2	N_D	K	I_S	λ(K)	λ(IS)
H_2	0.0674	0.0992	0.0493	0.0496	0.4713	0.4719
F_2	0.2888	0.6166	0.3229	0.3083	0.7538	0.7452
HF	0.0558	0.0788	0.0392	0.0394	0.4450	0.4455
N_2	0.3181	0.5305	0.0883	0.0884	0.5451	0.5453
C_2	0.9779	2.1869	0.2876	0.2734	0.7323	0.7231

Table S2. Comparison of different indexes and λ values of the molecules in the AE6 dataset.

	S_2	N_D	K	I_S	λ(K)	λ(IS)
SiH_4	0.2137	0.3011	0.0383	0.0376	0.4425	0.4405
S_2	0.8007	2.2580	0.2636	0.4234	0.7166	0.8066
SiO	0.2764	0.4387	0.0942	0.0731	0.5541	0.5200
C_3H_4	0.3657	0.5962	0.0383	0.0745	0.4423	0.5225
$C_2H_2O_2$	0.4678	0.7755	0.0546	0.0775	0.4833	0.5277
C_4H_8	0.2447	0.3527	0.0152	0.0441	0.3512	0.4582

Table S3. Comparison of different indexes and λ values of several atoms in the second row.

	state	S_2	N_D	K	I_S	λ(K)	λ(IS)
Be	^1S	0.4268	0.7295	1	0.2432	1	0.7022
	^3P	0.6931	2.0000	1	0.6667	1	0.9036
C	^3P	0.8029	2.1803	1	0.5451	1	0.8592
	^1D	0.8724	2.0455	1	0.5114	1	0.8456
N^+	^3P	0.7924	2.1591	1	0.5398	1	0.8571
	^1D	0.8985	2.1191	1	0.5298	1	0.8531
N	^4S	1.0397	3.0000	1	0.8000	1	0.9457
	^2D	1.0397	3.0000	1	0.8000	1	0.9457
O^+	^4S	1.0397	3.0000	1	0.8000	1	0.9457
	^2D	1.0397	3.0000	1	0.8000	1	0.9457
O	^3P	0.6931	2.0000	1	0.6667	1	0.9036
	^1D	0.6931	2.0000	1	0.6667	1	0.9036

Table S4. Comparison of different indexes and λ values of the transition states in DBH24 dataset.

**reactions**	S_2	N_D	K	I_S	$\lambda(K)$	λ(IS)
$OH + CH_4 \rightarrow CH_3 + H_2O$	0.4278	1.1243	0.0721	0.3748	0.5182	0.7824
$H + OH \rightarrow O + H_2$	0.7930	2.1606	0.4652	0.5401	0.8259	0.8573
$H + H_2S \rightarrow HS + H_2$	0.4305	1.1303	0.1917	0.3768	0.6617	0.7835
$H + N_2O \rightarrow N_2 + OH$	0.8101	1.8520	0.1848	0.2165	0.6557	0.6821
$H + ClH \rightarrow HCl + H$	0.4631	1.1943	0.3070	0.3981	0.7444	0.7943
$CH_3 + FCl \rightarrow CH_3F + Cl$	0.5597	1.4178	0.1484	0.4726	0.6207	0.8291
$Cl^- \cdots CH_3Cl \rightarrow ClCH_3 \cdots Cl^-$	0.0665	0.0977	0.0135	0.0366	0.3409	0.4375
$F^- \cdots CH_3Cl \rightarrow FCH3 \cdots Cl^-$	0.0739	0.1109	0.0154	0.0416	0.3524	0.4516
$OH^- + CH_3F \rightarrow HOCH_3 + F^-$	0.0692	0.1023	0.0110	0.0384	0.3240	0.4426
$H + N_2 \rightarrow HN_2$	0.6780	1.5598	0.1995	0.2228	0.6683	0.6871
$H + C_2H_4 \rightarrow CH_3CH_2$	0.3573	0.6120	0.0890	0.0893	0.5462	0.5466
$HCN \rightarrow HNC$	0.3862	0.6368	0.0897	0.0716	0.5472	0.5174
